# In vitro anticancer activity of hydrogen sulfide and nitric oxide alongside nickel nanoparticle and novel mutations in their genes in CRC patients

**DOI:** 10.1038/s41598-021-82244-x

**Published:** 2021-01-28

**Authors:** Zjwan Housein, Tayeb Sabir Kareem, Abbas Salihi

**Affiliations:** 1 Department of Biology, College of Science, Salahaddin University-Erbil, Erbil, Kurdistan Region 44002 Iraq; 2 Department of Medical Laboratory Technology, Health Technical College, Erbil Polytechnic University, Erbil, Iraq; 3 College of Medicine, Hawler Medical University, Erbil, Iraq; 4 Department of Medical Analysis, Faculty of Science, Tishk International University, Erbil, Iraq

**Keywords:** Cancer, Molecular biology, Physiology

## Abstract

This study was carried out to assess the impact of nickel nanoparticles (NiNPs) as well as scorpion venom on colorectal cancer (CRC) cells in the presence and/or absence of 5-fluorouracil (5-FU), hydrogen sulfide (H_2_S), and nitric oxide (NO) donors and to determine alterations in endothelial NO synthase (eNOS) and cystathionine γ-lyase (CSE) enzyme-producing genes in CRC patients. The IC_50_ of both H_2_S and NO donors, along with NiNPs, were determined. The CRC cells were treated for 24hrs, and the cytotoxic activities were assessed using the MTT test. Moreover, the apoptosis was determined after 24hrs and 48hrs using TUNEL assay. Furthermore, the mutations in the eNOS gene (intron 4, -786T>C and 894 G>T) and CSE gene (1364GT) were determined using direct sequencing. The IC_50_ values for sodium disulfide (Na_2_S) and sodium nitroprusside (SNP) at 24hrs treatment were found to be 5 mM and 10^−6^ M, respectively, while the IC_50_ value for 5-FU was reached after 5-days of treatment in CRC cell line. Both black and yellow scorpion venoms showed no inhibition of cell proliferation after 24hrs treatment. Furthermore, Na_2_S showed a significant decrease in cell proliferation and an increase in apoptosis. Moreover, a co-treatment of SNP and 5-FU resulted in inhibition of the cytotoxic effect of 5-FU, while a combination treatment of NiNPs with Na_2_S, SNP, and 5-FU caused highly significant cytotoxicity. Direct sequencing reveals new mutations, mainly intronic variation in eNOS gene that has not previously been described in the database. These findings indicate that H_2_S promotes the anticancer efficiency of 5-FU in the presence of NiNPs while NO has antiapoptotic activity in CRC cell lines.

## Introduction

In the world, colorectal cancer, generally known as colorectal adenocarcinoma, is the third foremost identified malignant tumour and the second most common cause of cancer death with variation within world regions^1,2^. Contemporary, there is insufficient data available about the prevalence and deaths of CRC in the entire Kurdistan region of Iraq^3^. The first report about CRC incidence in Kurdistan found an increase in annual incidence throughout 2007–2009^4^. According to a study accomplished in the Kurdistan region of Iraq, CRC is the top four most common cancer type for both genders, and the mortality rate was 8.6% compared to the worldwide estimated mortality which is 9.2%^1,5^.

Hitherto, the stage of CRC is the most vital prognostic factor for surviving this disease and the treatment procedures for CRC are mainly surgical resection as long as cancer has not spread to distant organs followed by chemotherapy if necessary^6,7^. Over the last three decades, 5-FU is used as a core chemotherapy drug to treat patients suffering from CRC^8^. Other treatments like radiotherapy^9^, ancillary anticancer medicines to prevent relapse^10^, and combination treatments^11^ are applied to treat patients in a more advanced stage of CRC. Nevertheless, all these therapy pathways have their drawbacks; therefore, innovative therapeutic approaches are requested urgently^12^. Hence, investigators focused on natural resources such as scorpion venoms that contain a blend of toxins and catalytic enzymes which exhibit anticancer activities, and therefore, are considered a potential candidate for developing new drugs that could enhance the effectiveness of conventional therapy and enable reduction of high doses of chemotherapy drugs intake^13^.

Recent researches are focused on tumour targeting through nanotechnology, which is a novel strategy and is a promising tool for the treatment of CRC^14^. Nanoparticles are small in size and therefore transport easily through the cell and could remain inside the cancerous cell and destroy it with limited damage to the healthy cells and tissues. This target-specific character is an important factor for developing nanodrug for the treatment of colorectal cancer; however, there are limited studies available that evaluate the cytotoxicity of nickel nanoparticles (NiNPs) on CRC cells^15^.

Hydrogen sulfide (H_2_S) and Nitric oxide (NO) are gaseous molecules known as "gasotransmitters"^16^; they have various roles in normal human physiology and regulation of cancer-related events such as proliferation, angiogenesis, cell apoptosis, and metastasis^17–19^. Different enzymes endogenously catalyze each of these molecules in various cell types^20^. H_2_S is mainly raised from the amino acids L-cysteine by two separate enzymes cystathionine γ-lyase (CSE) and cystathionine β-synthase (CBS). At the same time, NO is enzymatically catalyzed by one of the four major isoforms of NO synthases, namely neuronal NOS, inducible NOS (iNOS), and endothelial NOS (eNOS) and lately mitochondrial NOS from the amino acid L-arginine and oxygen^21,22^. H_2_S and NO reveal the exceptional capability to diffuse free through the cell membranes, accordingly acting as regulatory signalling molecules and it turns out to have a paradoxical effect in the cells, being either promotive or suppressive reliant on cell types and the concentration and which pathway is consequently activated^21,23^.

The findings in studies regarding the effect of NO and H_2_S on CRC cell death and related angiogenesis are ambiguous^24,25^. Hence, this study aims to find out the impact of venoms and nickel nanoparticles (NiNPs) on CRC cells treated with or without 5-FU, H_2_S, and NO donors or a combination of these treatments. Furthermore, to evaluate the genotypic variations and polymorphisms in eNOS and CSE enzyme-producing genes in CRC patients.

## Results

### Anticancer activity of scorpion's venom

**Figure 1:**
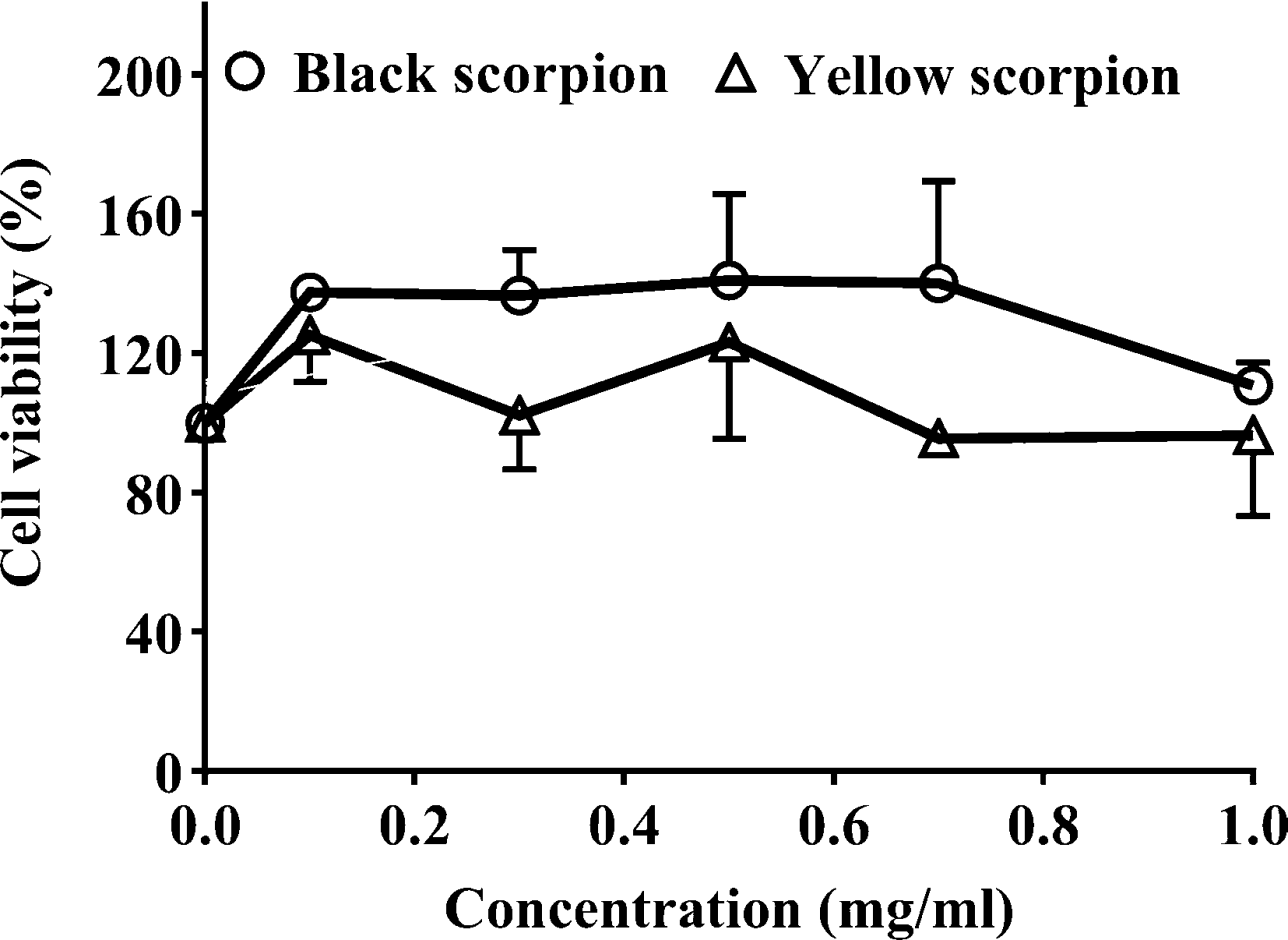
Cytotoxic effect of crude scorpion venom on CRC cell line. The cell viability after treatment for 24hrs on the HT-29 CRC cell line with *Hottentotta Saulcyi* scorpion venom (Black scorpion) and *Androctonus Crassicauda* scorpion venom (yellow scorpion), respectively.

The in vitro cytotoxic effect of crude black scorpion venom (*Androctonus Crassicauda*) and yellow scorpion venom (*Hottentotta Saulcyi*) was conducted in a dose-dependent manner using CRC cell line (HT-29). The indirect measure of cell viability was determined using MTT assay. Both scorpion venoms showed no inhibition of cell proliferation after 24hrs treatment (Fig. 1)**.**

### Anticancer activity 5-FU, Na_2_S, and SNP

The cytotoxic effect of 5-FU, Na_2_S, and SNP was determined in a dose-dependent manner to obtain an appropriate concentration of these agents for 50% cell death using MTT assay. This dose concentration represents the IC_50_ of that specific agent in the HT-29 CRC cell line after 24hrs of exposure. The cell viability of each drug or agent and after treatment with the particular drug or agent or a combination of treatment were assessed. The IC_50_ values for Na_2_S and SNP at 24hrs treatment were found to be 5 mM and 10^−6^ M, respectively. Furthermore, we confirmed that the IC_50_ value for 5-FU was 11.25 µM and reached after 5-days of treatment in the case of the HT-29 cell line.

**Figure 2:**
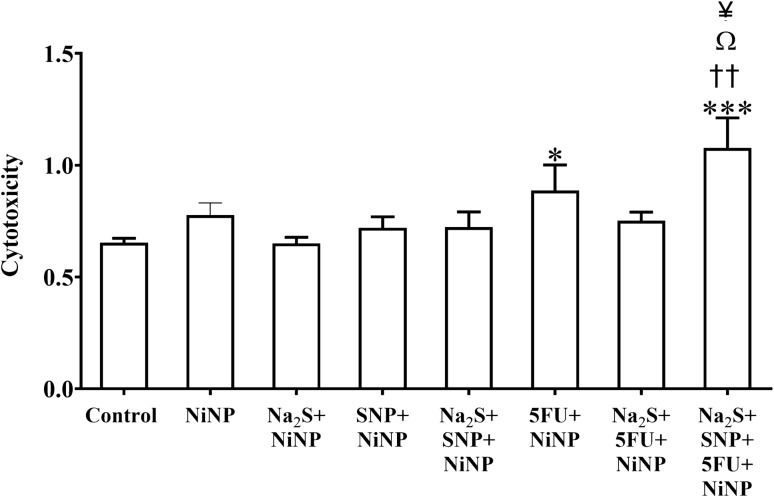
Cytotoxic effect of NiNPs on CRC cell line. The cell viability after treatment with NiNP for 24hrs on the CRC cell line (HT-29). The *, †, Ω and ¥ indicates statistical significance difference from control, Na_2_S+NiNP, SNP+NiNP, and Na_2_S, SNP+NiNP, respectively.

Next, we investigated the cytotoxic effect of these agents and the drug individually or in combination treatments on the HT-29 CRC cell line (Fig. 2). Our result presents the highly significant (*P* < 0.001) cytotoxic effect of 5-FU and a combination of 5-FU and Na_2_S compared to the control cells. At the same time, treatment with 5-FU and a combination of Na_2_S and SNP shows significant (*P* < 0.05) more cell death compared to cells treated with individual Na_2_S. Moreover, a co-treatment of Na_2_S and 5-FU shows a highly significant (*P* < 0.001) cytotoxic effect compared to cells treated with only Na_2_S.

A further data analysis showed that co-treatment of Na_2_S and SNP causes highly significant (*P* < 0.001) cytotoxicity compared to cells treated with only 5-FU, whereas co-treatment of 5-FU and SNP decreases (*P* < 0.01) the cytotoxic effect compared to 5-FU, but the effect remains significant.

Our data also shows that co-treatment of Na_2_S and SNP induces a highly significant (*P* < 0.001) cytotoxic effect compared to a co-treatment of 5-FU and Na_2_S. While there is a decrease in cell cytotoxicity in co-treatment of 5-FU, Na_2_S, and SNP (*P* < 0.05) compared to Na_2_S and SNP treated group. Finally, CRC cells treated with a combination of 5-FU and Na_2_S show a highly significant (*P* < 0.001) cytotoxic effect compared with the co-treatment of 5-FU and SNP.

### Anticancer activity NiNPs

**Figure 3:**
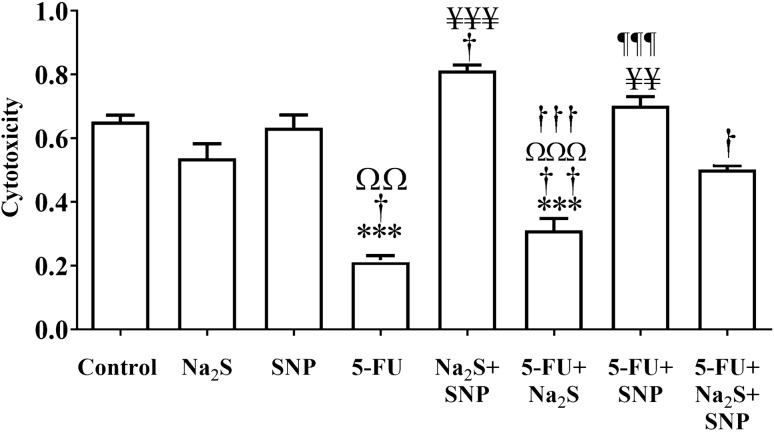
Cytotoxic effect of 5-FU, Na_2_S and SNP and combination of these treatment on CRC cell line (HT-29) after 24hrs. The *, †, Ω, ¥, † and ¶ indicates statistical significance difference from control, Na_2_S, SNP, 5-FU, Na_2_S and SNP, and 5-FU and Na_2_S, respectively.

We also investigated the cytotoxic effect of NiNPs on the HT-29 cell line using MTT assay for 24hrs (Fig. 3). Individual NiNP treatment has no cytotoxic activity in the HT-29 CRC cell line. In contrast, there is a highly significant increase (*P* < 0.001) in cytotoxicity in cells treated with a combination of H_2_S, SNP, 5-FU, and NiNP compared to the control group. The further data analysis showed that there is a significant increase (*P* < 0.01, *P* < 0.05, and *P* < 0.05) in cytotoxicity of cells co-treated with Na_2_S, SNP, 5-FU, and NiNP compared to co-treatments Na_2_S, NiNP; SNP, NiNP, and SNP; Na_2_S and NiNP, respectively.

### Antiapoptotic activity 5-FU, Na_2_S, and SNP

**Figure 4:**
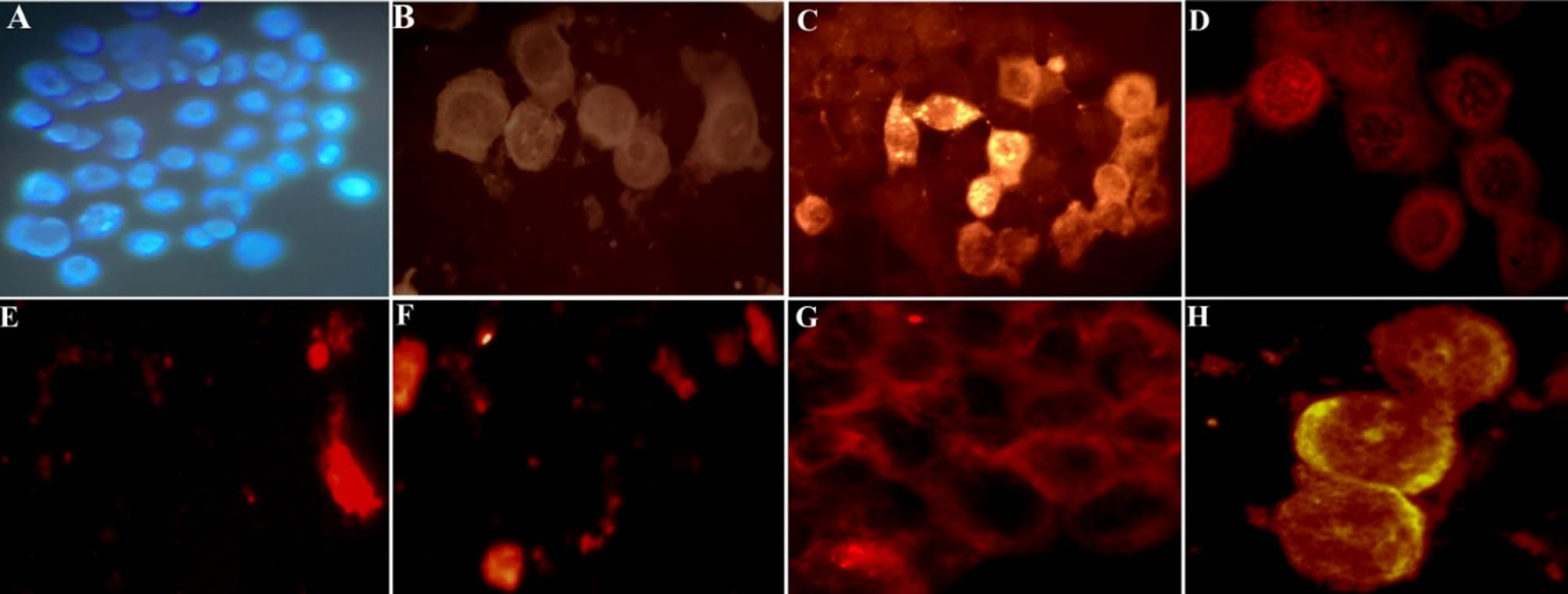
HT-29 CRC cell death detection by TUNEL assay after 24hrs. Apoptotic cells presented their morphology by taking up fluorescent dye. (**A**) The nucleus of untreated cells stained with DAPI (100 × magnification). (**B**) Control. (**C**) Cells treated with Na_2_S (IC_50_: 5 mM). (**D**) Cells treated with SNP (IC_50_: 10-6 M). E. Cells treated with 5-FU (11.25 µM after five days). (**F**) Cells treated with the combination of 5-FU and Na_2_S. (**G**) Cells treated with the combination of 5-Fluorouracil and SNP. (**H**) Cells with the triple combination of 5-Fluorouracil, Na_2_S, and SNP (images B-H at 1000 × magnification).

The DNA fragmentation results after 24 h incubation (Fig. 4) showed DNA fragmentation around 1–2%, in the control group, while the tunnel staining presented an increase of DNA fragmentation to 12% after treatment with Na_2_S. Besides, tunnel staining exhibited an antiapoptotic effect of CRC cells treated with SNP. Treating the cells with 5-FU causes total destroying of CRC cells after 24hrs, therefore only the debris of cells was visible in the slides. This result was almost the same for cells treated with both 5-FU and Na_2_S. In the presence of SNP agent, the cytotoxic effect of 5-FU is inhibited, and more CRC cells survived. The effect of a triple combination of 5-FU, Na_2_S, and SNP showed a distinctly decrease in cell survival, and more DNA fragmentation was visible.

**Figure 5:**
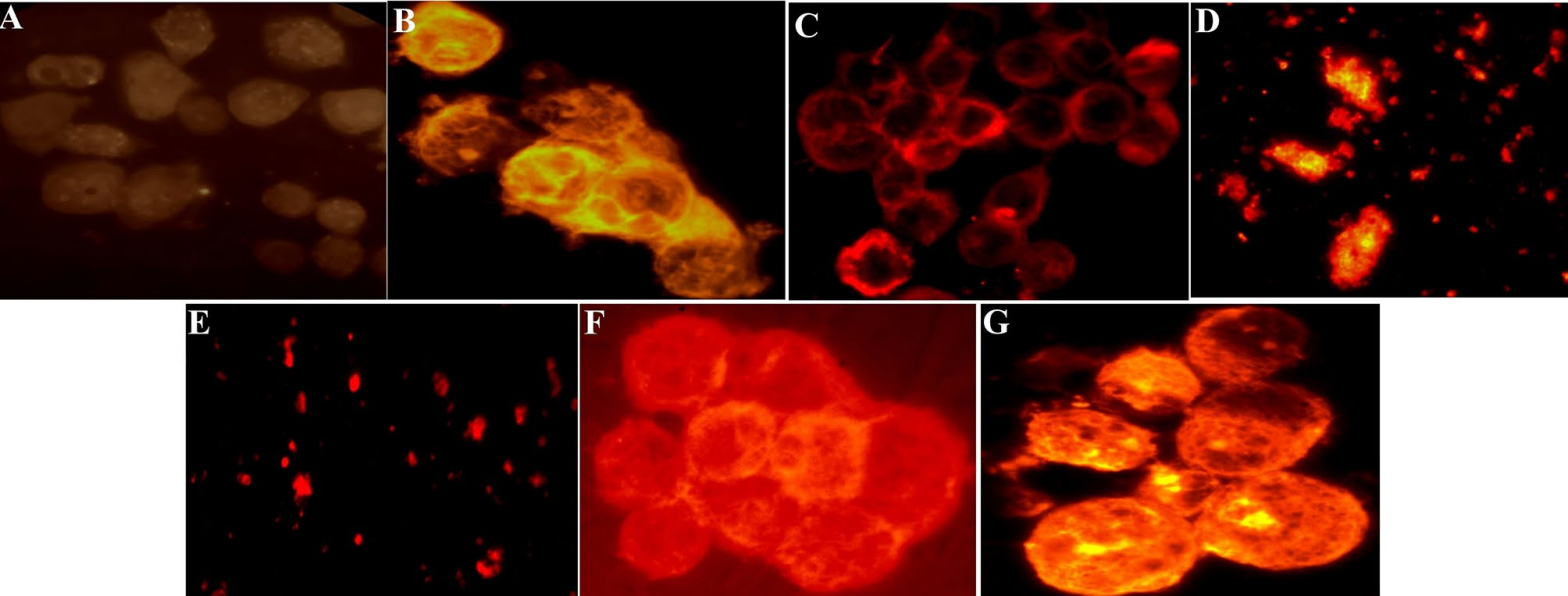
HT-29 CRC cell death detection by TUNEL assay after 48hrs. Apoptotic cells presented their morphology by taking up a fluorescent dye. (**A**) control. (**B**) cells treated with Na_2_S (IC_50_: 5 mM). (**C**) Cells treated with SNP (IC_50_. 10^−6^ M). (**D**) Cells treated with 5-FU (11.25 µM after five days). (**E**) Cells treated with the combination of 5-FU and Na_2_S. (**F**) Cells treated with the combination of 5-FU and SNP.G. Cells with a triple combination of 5-FU, Na_2_S, and SNP (images at 1000 × magnification).

We then investigated the DNA fragmentation after incubation of cells for 48hrs with Na_2_S, a slightly increased DNA fragmentation was shown compared to 24hrs exposure (Fig. 5).

### Genetic polymorphisms of eNOS and CBS

The result of gel electrophoresis in which the PCR product was separated in a 3% gel concentration, showed differences in the thickness of the amplicon. However, we were not able to distinguish between the heterozygous and homozygous variants. Therefore, we decided to genotype the three different single nucleotide polymorphisms in the eNOS gene and one in the CSE gene. Following DNA sequencing, we analyzed the mutations in eNOS and CSE gene using Mutation Surveyor (Table 1).

**Table 1:** Variants identified in CRC patients analyzed with mutation DNA variant analysis.

Gene	Chromosome position	Mutation	Mutation genotype	Heterozygous/homozygous	Variants	Amino Acid change	Molecular consequence	External database
**eNOS**								
VNTR4	7:150,694,330	A>AG	AG	Heterozygous	c.582+317A>G	N/A	Intron variant	gnomAD,dbSNP
	7:150,694,368	A > AC	AC	Heterozygous	c.582+355A > C	N/A	Intron variant	Not found
	7:150,694,369	G > GA	GA	Heterozygous	c.582+356G > A	N/A	Intron variant	Not found
	7:150,694,370	T > TG	TG	Heterozygous	c.582+357T>G	N/A	Intron variant	dbSNP
	7:150,694,373	Deletion A	-A		c.582+360delA	N/A	Intron variant	Not found
	7:150,694,373	A > AG	AG	Heterozygous	c.582+360A > G	N/A	Intron variant	Not found
	7:150,694,376	C > AG	AG	Heterozygous	c.582+363C > A+ 582+363C > G	N/A	Intron variant	Not found
	7:150,694,374	G > A	AA	Homozygous	c.582+361G >+ 582+361G > A	N/A	Intron variant	gnomAD,dbSNP
	7:150694378_ 7:150,694,387	del TGCTGCGGGG	–	–	c.582+365_ c.582+374 del TGCTGCGGGG	N/A	Intron variant	Not found
	7:150,694,375	A > AG	AG	Heterozygous	c.582+362A > G	N/A	Intron variant	Not found
	7:150,694,388	G > GC	GC	Heterozygous	c.582+375G > C	N/A	Intron variant	Not found
	7:150694394_ 7:150,694,395	insertion AGT CTA AAC CTA CTG CGG GGG TGA GGA	+	Heterozygous	c.582+381_c.582+382het_ins AGTCTAAACCTACTGCGGGGGTGAGGA	N/A	Intron variant	Not found
	7:150,694,389	T > TG	TG	Heterozygous	c.582+376T>G	N/A	Intron variant	genomAD,dbSNP
	7:150,694,633	Duplication A	++		c.582+620dupA	N/A	Intron variant	Not found
	7:150694394_ 7:150,694,395	insertion AGT CTA AAC CTG CTG CGG GGG TGA GGA	+	Heterozygous	c.582+381_c.582+ 382het_ins AGTCT AAACCTGCTGCGGGGGTGAGGA	N/A	Intron variant	Not found
**eNOS**								
rs2070744 (T-786C)	7:150,690,079	C > CT	CT	Heterozygous	c.51-762C > T c.51-762C > T	N/A	Genic Upstream Transcript Variant	dbSNP
		C > T	TT	Homozygous		N/A		dbSNP
**eNOS**								
rs1799983 (G894T)	7:150,696,111	T > G	TG	Homozygous	c.894T>G	(Glu298Asp)	Missense variant	dbSNP
**CTH**								
Rs1021737 (1364G-T)	1:70,905,057	G > GA	GA	Heterozygous	c.1218+247G > A	(Ser403Ile)	Missense variant Missense variant	Not found
	1:70,904,800	G > GT	GT	Heterozygous	c.1208G > T	(Ser403Ile)		dbSNP

In total, we validated 19 mutations in the 27-bp repeat polymorphism in intron 4 (27 bp-VNTR) of the eNOS gene at a different position on chromosome 7. The most nucleotide substitution that occurred in the 27 bp-VNTR was five times A→G. The variant mutation (c.582+317A>G) on chromosome position 7:150694330 has been previously described in external public databases such as dbSNP, gnomAD (Table 1). However, the A→G substitution in the mutation variants (c.582+360A>G, c.582+363C>A and c.582+362A>G) were not found in the external databases.

The T→G substitution (c.582+357T>G) on chromosome position 7:150694370 has occurred in two different CRC patients, and this mutation has been previously described; while, the T→G substitution with mutation variant (c.582+376T>G) that occurred on chromosome position 7:150694389, in which this mutation was not found previously in the databases mentioned above. However, the intronic nucleotide substitution A→C (582+355A>C) and G→A (c.582+356G>A) took place in the equal frequency of two, and G→C substitution (c.582+375G>C) turned out to occur only once in our patients that has not earlier been described in the databases.

Correspondingly, two deletions occurred in intron 4 including a single nucleotide deletion A (c.582+360delA) along with ten nucleotide deletions (c.582+374del TGCTGCGGGG). Furthermore, an heterozygous insertion of twenty-eight nucleotides occurred in intron 4 (c.582+381_c.582+382het_ins AGTCTAAACCTACTGCGGGGGTGAGGA) followed by a duplication of nucleotide A (c.582+620dupA). All these deletions and insertions found in intron 4, were not described previously in external databases. The G→A substitution that leads to homozygous mutation genotype were found in intron 4, and this mutation has been described in both dbSNP and gnomAD database.

**Figure 6:**
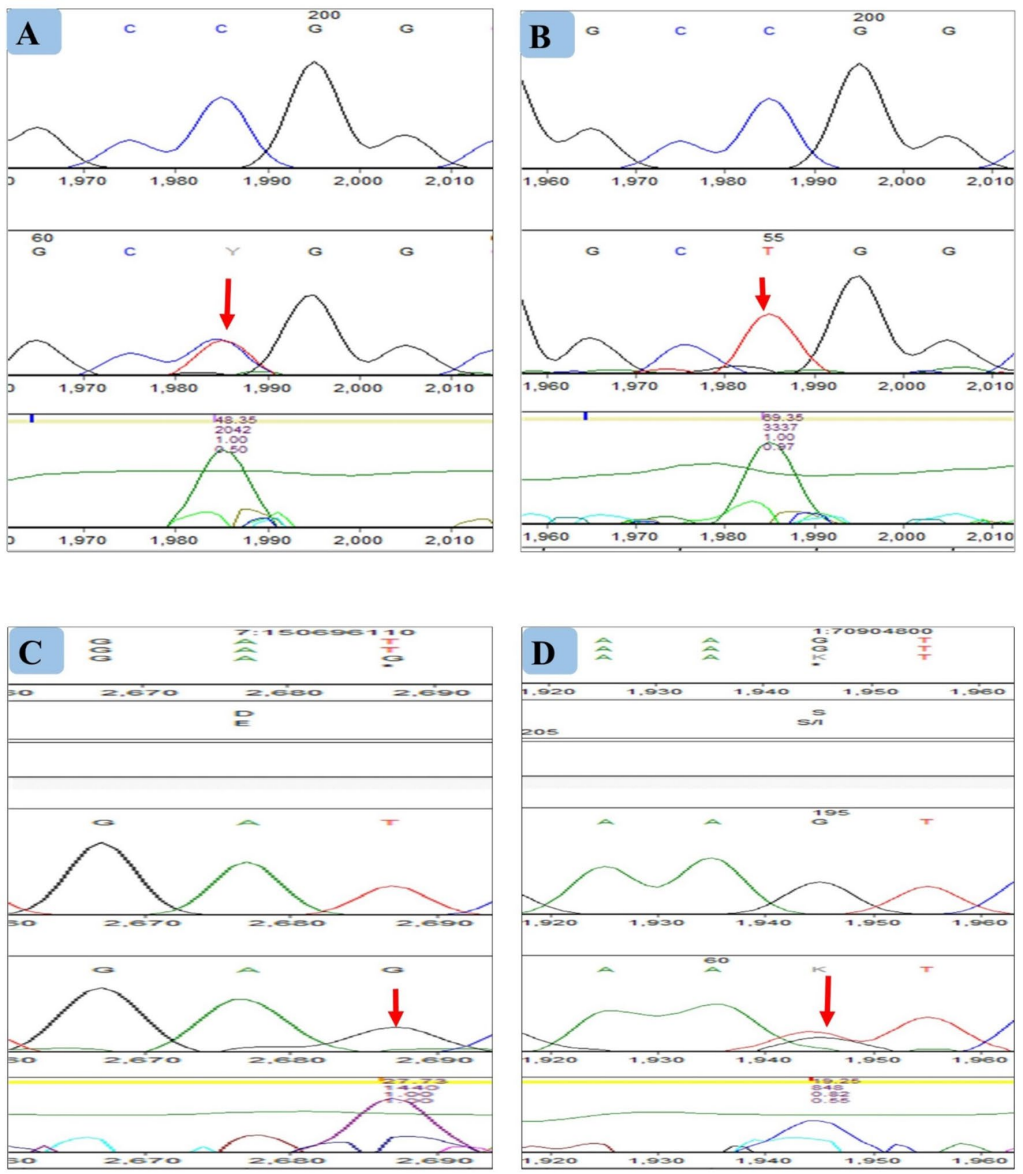
Graphical display of identified SNPs in eNOS and CSE gene (Sanger sequencing and analysis by mutation Surveyor software version 5.1. intronic mutation (VNTR4). Line 1 represents the forward reference trace Line 2 the patient DNA trace and line 3 the mutation trace and the arrow show the nucleotide substitution. (**A**) shows the heterozygous mutation -786T>C (c.-51-762C>T). (**B**) homozygous (T-786C) (c.-51-762C>T). (**C**) homozygous mutation in SNP G896T that leads to amino acid change (Glu298ASP) and (**D**) shows the mutation in SNP (364G-T) of the CSE gene that leads to amino acid change (Ser403Ile).

Alongside the Intronic mutations in the eNOS locus, we studied mutations in a T to C single nucleotide polymorphism (SNP) in the promoter region (T-786C, rs2070744). We found six times the heterozygous nucleotide substitution C>T (c.-51-762C>T) and two homozygous mutations that have been previously described in a public database (Table 1). The difference between the homozygous and heterozygous substitution is demonstrated in Fig. 6a,b, respectively. Besides, we determined the genotype of G→T (Glu298ASP; rs1799983) polymorphisms in exon 7 of eNOS. Further analysis of our data revealed that a missense mutation occurred in 2 out of 5 patients in which guanine is replaced by thymine at exon 7 that leads to a different codon that changes the amino acid Glutamate into Aspartate at codon position 298. This mutation is known in the external database, and this missense mutation is shown in Fig. 6c

Last but not least, we identified a common single nucleotide polymorphism, namely c.1364G>T in exon 12 of CSE, rs1021737 in CRC patients. The missense heterozygous SNPs (c.1208G>GT) on chromosome position1:70904800 causing a change in amino acid Ser to Ile at position codon position 1218 (Fig. 6d) and this mutation is earlier described in databases such as gnomAD, ClinVar, and dbSNP, while mutation variant (c.1218+247G>GA) that occurred on chromosome position 1:70905057 have not been found in database.

## Discussion

Colorectal cancer remains one of the most common cancers in humans with high incidence and mortality across the world. The existing conventional chemotherapeutic agents exhibit shortcomings due to the lack of selective targeting of cancerous cells. For this reason, researchers are working towards target therapy for the development of the most effective treatment.

For decades, scorpion venom has been used in traditional treatments, and recently researchers exhibited the anticancer properties of scorpion venom^26^. In this study, we examined the cytotoxic effect of the crude scorpion venoms, including *Androctonus Crassicauda* and *Hottentotta Saulcyi* on the CRC cell line (HT-29). The findings of this study showed no inhibition of cell proliferation after 24hrs treatment with high concentrations of scorpion venoms. This result is in contrast to previous studies wherein they showed cytotoxicity and cell cycle arrest of scorpion venom (*Androctonus bicolor*) on breast and CRC cell lines^27^. Moreover, Zargan et al. used *Androctonus crassicauda* scorpion, and their findings showed cytotoxicity and cell cycle arrest in the transformed breast cancer cell line^28^.

Furthermore, a variety of anticancer effects such as inducing apoptosis, cell cycle arrest, along with inhibition of invasion, metastasis, and angiogenesis in cancer cells, which are all targets of hallmarks of cancer alters when a variety of cell lines are treated with different bioactive peptides of scorpion venoms as reviewed recently^29^. The disability of both venom in this study to cause cytotoxicity could attribute to the venom preparation in this explicit investigational setup, where the venom is directly used after extraction from the scorpion without freeze-drying.

One of the main chemotherapeutic treatments for CRC includes 5-FU. The current result shows highly significant cytotoxicity of 5-FU on the HT-29 cell line. This is consonance with the recently published work by Attoub *e al*. wherein they demonstrate a reduction of different human CRC cells after treatment with 5-FU^30^. Nonetheless, the clinical application of 5-FU often causes chemoresistance. Therefore it is necessary to pursuit combination therapy to minimize the side effects that occur in patients^31^. The precise role of gasotransmitters H_2_S and NO in cancer remains debatable and demands further attention. Several studies have ascertained that NO and H_2_S have a dichotomous role in cancer, either promoting growth and proliferation. In contrast, others suggest antitumor effects such as inhibition of cell proliferation and suppression of DNA synthesis of the cancer cells as remarked by Kashefi in a recent review^32^. Given the positive role of these two gasotransmitters in the inhibition of cancer cell proliferation, in the present study, we have investigated the effect of NO and H_2_S donors individually or in combination with 5-FU on the HT-29 cell line for 24hrs.

Our data illustrates that Na_2_S significantly increases the cytotoxicity of HT-29 cells after combination with 5-FU compared to untreated cells. The findings in this study are in a similar vein with Oláh et al*.*^24^*,* who claim that using an increased level of H_2_S above the optimum concentration suppresses CRC cell proliferation. However, this result is not in line with Szabo et al.^33^, who depict that H_2_S stimulates tumour progression in the CRC cell line. In the same way as H_2_S, we examined the cytotoxic effect of NO donor SNP on the CRC cell line. The result shows no cytotoxicity of SNP on HT-29 cells compared to untreated cells while eradicates the cytotoxic effect of 5-FU. The latter findings might indicate that SNP acts as an antiapoptotic agent and probably interact with 5-FU and abolish the cytotoxic effect of 5-FU, which makes HT-29 cells resistant to 5-FU. According to a recent review, NO acts according to the bell-shape model in a concentration-dependent manner similar to H_2_S^24^. The interaction between H_2_S and NO that leads to the production of H_2_Sn has recently been studied and has shown that it could affect several physiological events such as activation of a particular tumour suppressor gene^34^. Therefore, it is important to study the effects of H_2_Sn in CRC cell lines and find the mechanism of action in future studies. Furthermore, the findings of a study that used a hybrid molecule containing nitric oxide and H_2_S combined with aspirin (NOSH-aspirin) which has anti-tumour properties demonstrated a synergistic effect with 5-FU treatment in xenograft model of colon cancer^35^.

To study the apoptosis induction from a different endpoint, we measured DNA fragmentation using the TUNEL assay. The results demonstrated a ten-fold increase in DNA fragmentation in HT-29 cells treated with Na_2_S compared to the untreated cells, while 100% of the cells were killed using 5-FU or co-treatment with Na_2_S and 5-FU. Our finding is in agreement with recently published in vivo work, wherein they demonstrate a significant increase in TUNEL positive cells after inhibition of CBS^36^. Earlier published work has shown CBS to be overexpressed and promote cell proliferation and stimulate angiogenesis in CRC patients^33^. These findings might propose the fact that these agents are acting as proapoptotic agents and could be a suitable candidate for future target therapy against CRC. In the meantime, treating HT-29 cells with SNP for 24hrs and 48hrs did not demonstrate any DNA breakage, while a co-treatment with 5-FU and SNP more cells survived. The latter finding is in line with our anticancer activity result wherein no effect of SNP was determined, and SNP neglected the cytotoxic effect of 5-FU. Besides, a triple combination treatment of 5-FU, SNP, and Na_2_S showed an increase in DNA fragmentation and a decrease in cell survival. Perhaps, H_2_S could be considered as a potent anticancer agent.

Nanoparticles have gained immense attention in the field of cancer therapy due to their small size and highly reactive and specific feature, and due to its target-specific character, it enables a personalized treatment as described in two separate reviews^37,38^. Having discussed the positive role of NO and H_2_S in colon cancer, we aimed to observe the anticancer activity of NiNP on HT-29 cells by measuring the cytotoxicity of the particles and in combination with 5-FU and the donors of these two gasotransmitters. Our result showed that there is no significant difference in cell survival after exposure of CRC cells to NiNP for 24hrs, while co-treatment of NiNP with 5-FU, Na_2_S, and SNP shows a significant increase in cytotoxicity. Of note, SNP does not inhibit 5-FU cytotoxicity. These findings are inconsistent with a recently published work where they demonstrate reduced cell viability and apoptosis induction after treatment with high concentration (600 µg/ml) Nickel oxide (NiO) NPs in CRC cell lines including HT-29^15^. Several studies are indicating that nanoparticles are used for enhancement of the efficiency of 5-FU, however, to our knowledge there are no NiNPs used in combination with neither 5-FU nor the agents H_2_S and NO and no data is available about the synergistic effect of NiNP^39^.

The recognition of targetable mutations determines current developments toward individualized treatment. One of the new treatments for metastatic CRC (mCRC) patients is the Cetuximab which is a monoclonal antibody that acts explicitly on the epidermal growth factor receptor (EGFR). This treatment is applied to mCRC patients without a mutation in the RAS gene. The reason for this is that CRC patients with mutations in this particular gene will fail to respond to this target-direct therapy^40^. Hence, a prescreen for the mutation before target therapy is mandatory.

In the current study, stage III and IV patients were screened for variants and polymorphisms in eNOS and CSE genes using a direct sequencing method. In this paper, the CSE gene will be reported as CTH according to the SNP database in NCBI. The eNOS gene has around168 polymorphism. We only analyzed three polymorphisms in eNOS genes and one polymorphism in the CTH gene that has been broadly studied in the literature. The genetic features of CRC patients are summarized in Table 1. The most prominent finding in our genotypic study is that two patients have 10 and 12 mutations in intron 4 (including single substitution, followed by duplication, large insertions as well as deletion). The current findings demonstrate the majority of heterozygous substitutions are A>G followed by CG and TG. However, this is not in line with the records found in the COSMIC database where more than 50% of the substitutions are G>A followed by G>T and T>A in the adenocarcinoma sample tissue of the colon cancer patients. Moreover, it is worth noting that our result showed the least mutations rate occurrence in 894G>T variant of eNOS and CTH gen.

To the best of our knowledge, the majority of detected mutations that occurred in selected CRC patients are not seen in the public database yet (Table 1). This might indicate that novel mutations in eNOS and CTH are validated on a specific location on a chromosome, that not previously have been described.

The findings regarding the involvement of eNOS polymorphism in cancer are controversial, likely due to considered factors such as analyzed sample size, cancer type, and the ethnic-dependent variation^41^. A meta-analysis study reported a significant association of eNOS intron 4, and -786T>C polymorphism in overall cancer and 894 G>T polymorphisms with the risk of breast cancer^42^. While another conducted meta-analysis study reported a conflicting result wherein they did not found a significant association of SNP 894 G>T with overall cancer susceptibility^43^. Moreover, Ulivi and his colleagues claim to have identified a specific haplotype in eNOS polymorphism (+ 894GG and VNTR4) that are significantly associated with the overall survival of mCRC patients. Therefore, the researchers consider using this identified eNOS variant to predict the efficiency of bevacizumab-based chemotherapy in mCRC patients^44^, and a Chinese study confirmed a significant association between the intron 4 variant and increased cancer risk in Taiwanese individuals under the age of 60 years^45^. One of the limitations of this study is the restricted number of analyzed samples and SNPs used in this study that limits the evaluation of the results.

## Conclusions

These findings reveal that H_2_S could be used for enhancement of the anticancer efficiency of 5-FU in the presence of NiNPs in the CRC cell line, while NO could be considered as an antiapoptotic agent that enables HT 29 cells resistant to 5-FU treatment. Moreover, changing the experimental setup in future research by administrating loaded NPs or venom or a combination with H_2_S in liposomes might deliver the agents more efficiently to the CRC cells leading to a better strategy of cancer treatment. Further investigation on the molecular level is essential to understand the mechanism by which the mutations in eNOS and CSE enzyme-producing genes perhaps could modify the role of H_2_S and NO in CRC patients.

## Materials and methods

### Cell culture

The human colorectal adenocarcinoma (HT-29 cells, IBRC C10097) were purchased from the National Cell Bank in the Iranian Biological Resource Center. These cells were grown as a monolayer in a 25 cm^2^ culture flask with Dulbecco's Modified Eagle Medium (DMEM high glucose, EuroClone cat: ECM0728L) containing L-glutamine. The culture media were supplemented with an antibiotic solution containing 1% (100×) Penicillin/Streptomycin (Capricorn Scientific Cat: PS-B) and 10% heat-inactivated FBS (Capricorn Scientific Cat: FBS-GI-12B) as the provision of nutrients. The cells were maintained at 37 C^o^ in a 5% CO_2_ humidified incubator (RS Biotech Galaxy Model R +, USA). The growth medium was changed every three days, and the viability of cells was assessed by direct observation of cells using an inverted phase-contrast microscope (Labomed, USA). The HT-29 cells were sub-cultured using 0.05% trypsin/EDTA (Capricorn Scientific cat: TRY-1B). Cell viability was assessed by staining 100 µl of cell suspension with trypan blue (0.2%), and cells were counted using a hematocytometer. All the experiments were performed using cells from passage 20 or less.

### Cell morphology analysis

To find out the IC_50_, the cells were treated with various concentrations of Na_2_S, an exogenous H_2_S donor, ranging from (1-6 mM), SNP a NO donor, with following concentrations (3 × 10^–5^–3 × 10^−8^ M) for 24hrs and 5-FU (11.25 µM) for 24, 48, 72hrs and 96hrs which expect to reach the IC_50_ after five days^46^. The morphological changes were examined using an inverted phase-contrast microscope (Labomed, USA) followed by MTT assay.

### In vitro cell proliferation assay

The reduction of (3-(4,5-dimethylthiazole-2-yl)-2,5-diphenyl-2H-tetrazolium bromide) is a method to measure and monitor cell proliferation. MTT assays were performed using the Cytoselect MTT Cell Proliferation Assay (Cell Biolabs, Inc., Cat: CBA-252, San Diego, CA, USA). After reaching 85% confluence, the HT-29 cells were trypsinized, and the cells were suspended in the growth medium, 2 × 10^5^ cells/well were seeded in a 96-well culture plate and incubated at 37 C° in a humidified 5% CO_2_ allowed to attach for 48hrs. The cytotoxicity of the compounds was determined in a dose-dependent manner to obtain the proper concentrations of the compound for 50% inhibition of cell growth (IC_50_). The growth medium was then changed with the new medium and exposed with IC_50_ concentration of the compounds and exposed for 24hrs at the following IC_50_ concentrations: Na_2_S (5 mM), SNP (1.10^–6^ M), 5-FU (11.25 µM, after five days), and the combination of these compounds and each condition is tested in 6 replications. After the incubation periods, 10 µl of Cytoselect MTT cell proliferation assay reagent was added to each culture well, and the 96-well plates were incubated at 37 C^o^ for 4hrs until a purple precipitate is visible. 100 µl of a detergent solution containing dimethylsulfoxide (DMSO) was added to each well to dissolve the purple formazan product. Following 2hrs of incubation at room temperature in the dark, the absorbance was determined at a wavelength of 630 nm by an Elisa reader (BioTek Instruments, Inc., USA).

### Preparation of Nickel nanoparticle suspension

Nickel nanoparticle with 40 nm in diameter size from (Sigma-Aldrich Co, St Louis, USA) was tested in this study. The method described by Abdulqader and Aziz^39^ was used to prepare NiNPs with some modification. Hence, a 1 mg/ml stock solution in the growth medium was prepared, and subsequently, the suspension was vortexed and then sonicated for 30 s with an ultrasonic homogenizer (150 VT model, constructed by Biologica, Inc., Manassas, VA, USA). Next, the suspension with nickel particles was placed on ice for 15 s, and, sonicated again for 3 min on ice. The NiNPs were vibrated for 2 min, immediately before the actual incubation to avoid agglomeration. The HT-29 cells were incubated with NiNP suspension with the following concentrations (0.1–30 µg/ml) for 24hrs, and the IC_50_ of (10 µg/ml) was determined. The IC_50_ of NiNP was subsequently used for treating the cells with NiNP alone or co-treatment with IC_50_ concentration of the following agents, including, 5-FU, Na_2_S, and SNP.

### Cell apoptosis assay

The terminal deoxynucleotidyl transferase (dUTP)-nick end labelling (TUNEL) method that determines DNA-strand breaks throughout the apoptosis process was used to confirm HT-29 cells undergoing apoptosis after treatment with different compounds for different time of period. Cell Meter TUNEL (Red fluorescence) Apoptosis Assay Kit (AAT Bioquest Inc., USA, Cat: 22,844) was used according to the manufacturer's suggestion procedure with some modifications. In short, 6 × 10^4^ of HT-29 cells were grown for 72hrs on coverslips then treated with or without IC_50_ of the following compounds; Na_2_S (5 mM), SNP (10^–6^ M), 5-FU (11.25 µM, after five days), and the combination of these compounds for 24 h, 48 h, and 72 h, 96 h, and 105 h at 37 °C with 5% CO_2_. After incubation coverslips were rinsed twice with ice-cold PBS (pH 7.4) followed by incubation with TUNEL kit reaction solution (consisting of 100× Tunnelyte Red 0.5 µl+ reaction buffer 50 µl) for 45 min at 37 C^o^ in a dark chamber and additional 4′,6-diamidino-2-phenylindole (DAPI) stain was conducted to identify the cell nuclei. Cells with red nuclei are TUNEL-positive indicating apoptosis and apoptotic cells were visually observed using a fluorescence microscope (Euromex, Holland) equipped with sCMEX-20 Microscope Camera (Euromex, Holland). The images of total cell nuclei stained with DAPI and CRC cells stained with TUNNEL fluorescent dye were captured at 100 × and 1000 × magnification, respectively. The quantity of TUNEL positive cells was given as a percentage of total cells counted in a minimum of six random microscopic fields.

### Collection of scorpions and their venom

The scorpion species (*Androctonus Crassicauda* and *Hottentotta Saulcyi*) were collected from different regions of northern Iraq by a professional researcher. The scorpion collection and venom isolation were carried out in accordance with the Guide for the Care and Use of Laboratory Animals and approved by the Animal Research Committee of Salahaddin University-Erbil. These arachnids were kept individually within the desirable condition and sustained with mealworms and water ad libitum. The scorpion venoms were ejected by electrical stimulation and directly used to expose the HT-29 cells with venom concentrations ranged from (0.1–1.0 mg/ml) for a period of 24hrs.

### Human CRC specimens

CRC specimens were collected from 35 patients at different hospitals in Erbil, Iraq (PAR, CMC, Rezgary, Zheen international hospitals). This study was approved by the Human Ethics Committee of the College of Science, Salahaddin University-Erbil. The study was conducted according to the criteria set by the declaration of Helsinki, and the signed informed consent forms were obtained from the participants approving to investigate the tissue material. All CRC were histologically confirmed, and the tissue specimens were snap-frozen in liquid nitrogen and stored at -80 C^o^ until DNA extraction.

### DNA extraction and quantification of DNA concentration

Genomic DNA was purified from CRC tissues following the manufacturer's protocols using the Genomic DNA Mini Kit for Tissue (Geneaid Biotech Ltd, Korea). The eluted DNA was assessed for concentration (A260) and purity (by the measure of the A260/A280 ratio) on Nanodrop (Thermo Fisher Scientific, UK). The Nanodrop instrument was blanked against the elution buffer, and the interpretation of DNA purity was based on an optimal A260/A280 ratio of 1.8.

### Genotype determination

In this study, we selected three commonly studied variations in the eNOS gene and one CSE variant, also known as CTH, located on chromosome 7 (7q36.1) and chromosome 1 (1p31.1), respectively. We genotyped 25% of the collected sample tissues for eNOS genetic polymorphisms rs2070744 (−786T>C) at the promoter region and 14% of the patient tissues for rs1799983 (894G>T), and 11% of the patient tissues for *intron* 4 27-base repeat variant (VNTR4). Furthermore, 14% of the genetic polymorphism rs1021737 of the CSE gene (1364GT) variant at exon 12 is genotyped.

First, the purified DNA was separately amplified for each genetic polymorphism by polymerase chain reaction (Techne TC-512, UK) using the following primers: eNOS (−786T>C) forward, 5′AAGGC AGGAGACAGTGGATGGA-3′ and reverse, 5′-CCCAGTCAATCCCTTTGGTGC TCA-3′. eNOS (894G>T) forward, 5′-TGGAGAGTGCTGGTGTACCCCA-3′ and reverse, 5′-GCC TCCACCCCCACCCTG TC-3′. eNOS (VNTR4) forward, 5′-AGGCCCTATGGTAG TG CCTTT-3′ and reverse 5′-TCTCTTAGTGCTGTGGTCAC-3′ and CSE (1364 G>T) forward, 5′-GGAC TTCTTGAGGAGTTGAAGC-3′ and reverse, 5′-ATTCTCACCTCCTTCAGAGGC-3′. The PCR thermocycling conditions included an initial denaturation step at 95 °C for 5 min, followed by 35 cycles of 95 °C for 30 s; different annealing temperatures were used for each polymorphism (-786T>C at 56 °C, 894G>T at 55 °C, VNTR4 at 55 °C and CSE 1364 G>T at 50 °C) for the 30 s; elongation at 72 °C for 1 min, and a final extension step at 72 °C for 5 min. All the PCR products were separated on 3% agarose gel electrophoresis and compared with the 50 bp DNA marker (Mini Sizer, Norgen Biotek Corp. Canada., cat:11200) and stained with safe dye (Safe gel stain Dye, Add Bio) before casting into the tray and visualized using a gel documentation system (UV transilluminator UST-20M-8K, Biostep GmbH, Germany).

Following the PCR procedure product was sent for sequencing using the same forward primers for each particular polymorphism by using automatic ABI PRISM 3130 DNA sequencer (Applied Biosystems, USA). Analysis of Sanger sequencing data was achieved using the Mutation Surveyor software package 5.1 (Soft Genetics), and the mutation result was compared with public databases, including gnomAD, dbSNP, ClinVar, and COSMIC).

### Statistical analysis

The results were analyzed using GraphPad Prism 6. One-way ANOVA with Tukey's multiple comparisons test was conducted to evaluate the statistically significant differences between the anticancer activity of different agents and untreated cells. All the experiments were carried out 3 to 6 times and the data were stated as mean ± SE difference.
